# Wide Local Excision of Complex or Infected Pilonidal Sinus Followed by Negative Pressure Wound Therapy: Does It Enhance Wound Healing?

**DOI:** 10.7759/cureus.48049

**Published:** 2023-10-31

**Authors:** Rohin Kansal, Ayushi Garg, Baninder Arora, Carol Singh, Kashish Malhotra, Madhav Mehta, Anubhavv Gupta, Harsh Kishore, Himel Mondal, Ashvind Bawa

**Affiliations:** 1 Department of General Surgery, Dayanand Medical College and Hospital, Ludhiana, IND; 2 Department of Internal Medicine, Dayanand Medical College and Hospital, Ludhiana, IND; 3 Department of Medicine, Adesh Institute of Medical Sciences and Research, Bathinda, IND; 4 Department of Research, Dayanand Medical College and Hospital, Ludhiana, IND; 5 Department of Physiology, All India Institute of Medical Sciences, Deoghar, Deoghar, IND

**Keywords:** pilonidal sinus disease, npwt, negative pressure wound therapy, daily dressings, vacuum-assisted wound closure, postoperative care

## Abstract

Background

Pilonidal sinus disease (PSD) is a chronic skin condition caused by hair retention that affects the sacrococcygeal cleft. The purpose of this study is to compare the efficacy of negative pressure wound therapy (NPWT) to routine daily dressings (DDs) in wound healing after complex or infected pilonidal sinus tract excision.

Materials and methods

The study included 81 individuals who had extensive local excisions for pilonidal sinuses that were complex or infected. Randomly selected participants were given either NPWT or the usual dressing. Between the two groups, the length of hospitalization, the amount of time needed to resume daily activities, and the amount of time needed for full wound closure were compared.

Results

Forty-two patients received NPWT, while 39 patients received DDs as usual. There was no discernible difference between the two groups in terms of operating time or hospital stay. However, patients who underwent NPWT experienced a quicker final wound closure (59.24 ± 10.21 days compared to routine DD with a mean time of 75.31 ± 14.68 days, P = 0.001) and returned to normal activities earlier (17.36 versus 21.75 days in NPWT and routine DD, respectively).

Conclusion

Compared to patients who received standard DDs, those who were postoperatively managed with NPWT showed faster wound healing and return to normal activities. Whenever feasible, this strategy may be employed to improve patient recovery.

## Introduction

Pilonidal sinus disease (PSD) is a recurring skin condition that frequently affects males and those with abundant body hair and results in a cyst or abscess in the crease between the buttocks [1]. The likelihood of contracting the disease can be increased by obesity, inactivity, and prolonged periods of sitting [2]. It has a bimodal incidence, manifesting most severely before puberty or after 60 years of age, with a higher rate seen in males [3]. PSD becomes more challenging as the sinus passage infiltrates deeper into the tissue, which may lead to abscesses and recurrent infections [4]. Complex PSD encompasses individuals who experience repeated infections or exhibit an excessive degree of branching within the sinus system. Also, post-operative pilonidal sinuses with abscesses can be included under the gamut of complex PSD. Complex PSD can be treated using various techniques, including surgical and non-surgical alternatives. The primary objective of the treatment is to remove the sinus tract and prevent recurrences [5]. Surgery options for complex PSD that leave the wound open include wide excision and marsupialization. Wide excision involves removing the entire sinus tract and surrounding tissue, which is considered the most effective treatment. Marsupialization consists of creating a small skin opening to allow the sinus to drain and keeping the opening open with sutures or a skin flap [6]. Additional protocols may be implemented after surgery to ensure recovery and prevent a recurrence, including antibiotics and daily wound dressing to prevent further infection. A significant dilemma posed to the surgeons is whether to close the wound or leave it open for secondary healing [6]. Initial wound closure is expected to be more pertinent than open wound healing. A recent review of the literature on wound closure for PSD has compared the efficacy of primary closure to that of secondary intention healing (open healing) [6, 7].

Primary closure results in quicker healing rates and a quicker return to work, but at the cost of an increased chance of recurrence. Comparing recovery by primary closure to healing by secondary intention, the likelihood of a pilonidal illness recurrence was reduced by 58% [8, 9].

Negative pressure wound therapy (NPWT) is a procedure that employs vacuum technology to remove excess fluids, bacteria, and debris from a wound while stimulating the formation of new tissues, aiding healing, and minimizing the risk of infection. NPWT refers to wound healing technology consisting of three major parts: a wound dressing, covers, and a pump. Wound dressing aids in transferring pressure from the pump to the wound itself, and modern NPWT typically utilizes reticulated open-pore polyurethane foam intended to equalize the negative pressure across the entire wound surface. NPWT is being used more and more on closed incisional wounds as a preventative measure to stop surgical site complications [10], as well as on wounds healing by secondary intention (left open to heal from the bottom up), such as chronic or infected wounds [11]. The present study aimed to assess the clinical effectiveness of NPWT with respect to hospital stay, wound closure, and return to regular activities after excision of the pilonidal sinus.

## Materials and methods

Settings and participants

This was a prospective study with systemic intervention allocation to different individuals. Patients who came to our tertiary care center for surgical treatment of complex or infected PSD were included in this study after providing written informed consent. The Institutional Ethics Committee of Dayanand Medical College and Hospital approved this study (DMCH/R&D/2022/156). Patients with simple PSD, those who underwent flap surgery, or those with a pilonidal sinus situated less than 3 cm from the anus were excluded from the study.

Management protocol

MRI of the sacral area was performed to gauge the full scope of the sinus tract. The perianal area was depilated prior to surgery, and the patient was instructed to keep the hair in that area short to avoid recurrences in the future. A wide local excision was performed on these individuals, extending to the pre-sacral fascia to remove the entire pilonidal tract. The wound was dressed at regular intervals for the first two postoperative days, according to our standard daily dressing (DD) procedure. Negative pressure wound therapy and DD were discussed with the patients as treatment possibilities. Patients were divided into groups based on their preferences. Following that, patients in the NPWT group had vacuum-assisted dressing, whereas others remained with their normal DD practice.

Routine daily dressings

The incision was kept open following the surgical excision. Patients were told to clean the wound daily for the first two weeks following the excision. Unless the wound was dirty and retained pus, no special dressings were needed. Patients were trained to remove the dressing, cleanse the area with saline, and do redressing at home. They were followed up regularly. The following directives were given to patients: (a) It is important to wash the wound after every bowel movement; (b) The dressing must always be kept dry; (c) The dressing has to be changed right away if it gets dirty or wet; (d) To guarantee that the patient completes a hygiene regimen at least once per day, it was advised to plan a shower time as soon as possible following a bowel movement; (e) The inside of the wound should be carefully flushed out, and soap, shampoo, and loose hair should be directed away from the afflicted region using a handheld sprayer. Infected wounds are irrigated with saline to remove any debris.

Negative pressure wound therapy

After two days of surgical excision, a surgeon or wound care nurse applied the NPWT. A sponge was used to clean the area first. A skin adhesive was used to prevent skin injury. NPWT consisted of foam coated by an adhesive semipermeable dressing. The wound was subjected to a constant negative pressure of 125 mm Hg for five days using a vacuum pump (NPWT unit). Patients were sent home with NPWT and were instructed on how to conduct routine checks to ensure the device's proper functionality. Additionally, they were advised to promptly report any changes or malfunctions in its operation to the wound care nurse. Consistent follow-up care and wound washing were provided between NPWT cycles. The process was repeated until the wound bed was prepared.

Post-wound bed preparation

Both groups of patients had their wound beds adequately prepped before being given a variety of wound closure options, such as skin grafting, secondary suturing, or DD, all of which encourage epithelization. The advantages of choosing skin grafting or secondary suturing, which might lead to a quicker recovery period, were fully explained to the patients. In addition, the length of the hospital stay after the surgery was recorded. To measure the pain intensity, the visual analog scale was used, and the patients were asked to rate their pain from 1 to 10 two weeks after the operation. All the patients were asked whether they were satisfied with the surgical procedure [12].

Data analysis

The analysis of the data began with a descriptive breakdown of various patient-related aspects, including demographic information, wound size, hospital stay, post-surgical discomfort, time taken to resume daily activities, and duration of wound healing. Results were presented using range, mean, SD, number of instances, and relative frequencies expressed as percentages. Binomial and Chi-square tests were used to compare categorical data, with a p-value of less than 0.05 considered statistically significant. Statistical computations were performed using IBM SPSS version 26 (IBM Corp., Armonk, NY, USA).

## Results

Patients' characteristics

**Figure 1:**
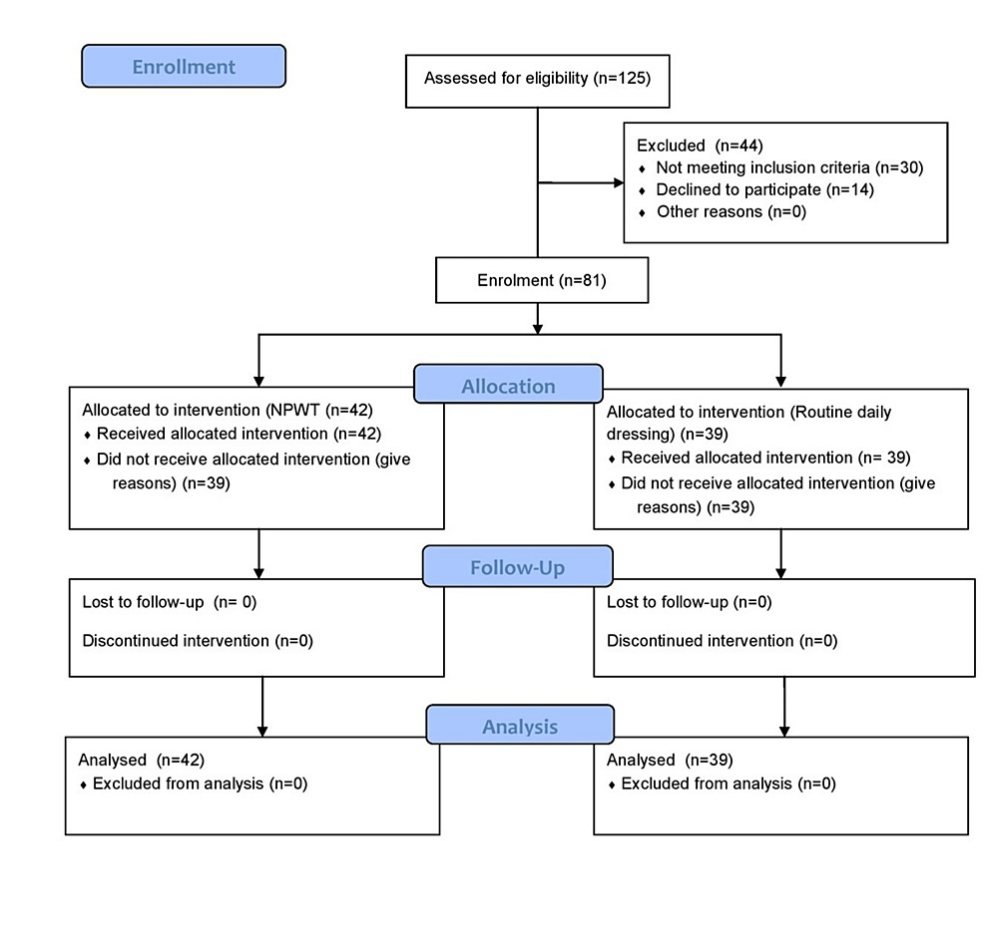
Flow diagram of the enrollment process.

A total of 81 patients were included in the study (Figure 1).

There were 64 (79.01%) men and 17 (20.98%) women. The mean age of patients was 26.40 ± 5.67 years (range 18-40 years) for the NPWT group and 24.46 ± 5.11 years for the routine DDs group. The demographic data are shown in Table 1.

**Table 1:** Comparison of variables between negative pressure wound therapy and routine daily dressing groups. NPWT: Negative pressure wound therapy. †P-value of Chi-square test *P-value of Mann-Whitney U test

Variable	NPWT (n = 42)	Routine daily dressings (n = 39)	P-value
Male to female ratio (m/f)	33/9	31/8	0.919†
Age (years) (mean±SD)	24.46 ± 5.11	26.40 ± 5.67	0.110*
Operative time (minutes) (mean±SD)	24.33 ± 5.45	24.77 ± 5.27	0.649*
Hospital stay (days) (mean±SD)	3.17 ± 1.34	3.64 ± 1.48	0.117*
Pain score after two weeks (mean±SD)	2.29 ± 1.27	3.82 ± 1.55	0.001*
Time to return to daily activities(days) (mean±SD)	17.36 ± 4.11	21.74 ± 3.53	0.001*
Time to wound bed preparation (days) (mean±SD)	35.26 ± 8.22	53.0 ± 13.67	0.001*
Complete wound closure time (days) (mean±SD)	59.24 ± 10.21	75.31 ± 14.68	0.001*

Of these patients, 25 had a history of surgery for PSD at other hospitals. Fourteen patients (17.2%) had undergone definitive surgeries, such as excision with primary closure or excision with healing by secondary intention, and 11 (13.5%) had only undergone drainage procedures and no excisional surgery. These patients re-presented some years after these procedures into the current patient group. NPWT was chosen by 27 patients with infected PSD, while 20 patients with infected PSD chose DD. The average area of the wound following excision in patients belonging to the NPWT group was 50.5 ± 14.82 cm2, whereas in the DD group, it was 48.83 ± 11.16 cm2. The statistical analysis revealed an insignificant difference between the two groups, with a p-value of 0.551. The average depth of the patients in the NPWT group was 2.09 ± 0.35 cm, while in the DD group, it was 1.94 ± 0.42 cm (p-value = 0.081).

Hospital stay

The mean duration of hospital stay did not differ significantly in either group. The NPWT group spent an average of 3.17 days, and the routine DD group stayed 3.64 days (p-value = 0.117) (Table 1).

Pain

On a pain scale of 10, patients receiving NPWT had a lower average pain score of 2.29 ± 1.27 compared to the routine DD group, which had a score of 3.82 ± 1.55 (p-value < 0.001) (Table 1).

Time to resume daily activities

Patients who had NPWT were able to resume regular activities sooner, with a median time of 16.5 days (interquartile range: 13.75-21.0 days), compared to the standard DD group, which had a median time of 22 days (interquartile range: 18.0-25.0 days) (p = 0.001).

Time to wound healing

**Figure 2:**
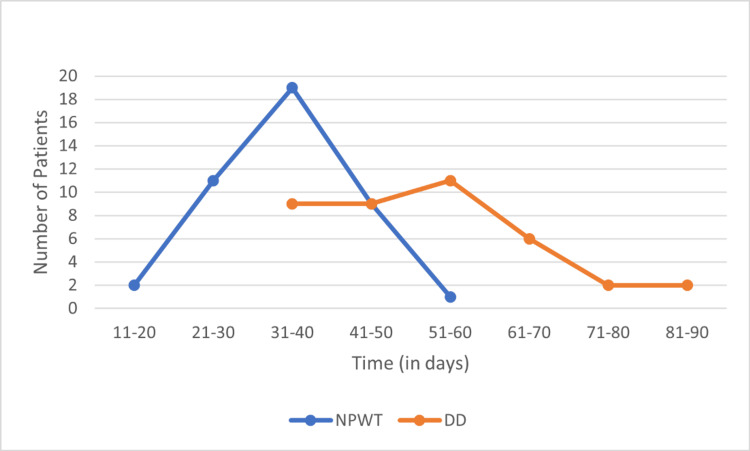
Time to wound bed preparation in patients after negative pressure wound therapy (NPWT) and daily dressing.

**Figure 3:**
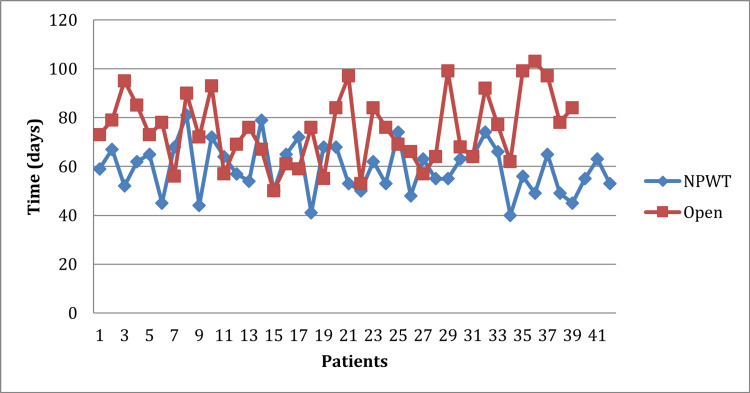
Comparison of time to complete wound healing after negative pressure wound therapy (NPWT) vs. daily dressing.

The median time to wound bed preparation in the group receiving NPWT was 36 days (range: 18-51) compared to 52 days (range: 32-84) receiving routine DD. Complete wound healing following surgery was accomplished in the NPWT group at an average of 59.2 days as opposed to 75.3 days in the control patients (p = 0.001). On average, the duration of follow-up for patients in the NPWT group was 74.95 ± 10.86 days, while for the DD group, it was 90.05 ± 14.35 days (p-value < 0.001). A comparison of time to complete healing can be seen in Table 2 and Figures 2-3. 

**Table 2:** Number of patients and time to complete wound closure. †P-value of Chi-square test *P-value of Mann-Whitney U test

		NPWT	Daily Dressing	P-value
Number of patients	Epithelization	27	27	0.895†
	Secondary Suturing	10	8	
	Skin Grafting	5	4	
Time to complete closure (days)	Epithelization	63.67 ± 9.0	78.14 ± 13.7	0.001*
	Secondary Suturing	49.40 ± 4.78	60.25 ± 7.67	0.002*
	Skin Grafting	55.0 ± 9.66	86.25 ± 11.29	0.003*

Complications

The rate of complications in both groups is comparable and not statistically significant, as indicated by a p-value of 0.829 obtained through a chi-square test. A comparison of complications among the groups can be found in Table 3.

**Table 3:** Complications in patients using NPWT compared to daily dressing. NPWT: Negative pressure wound therapy.

		NPWT	Daily Dressing	Chi-square value	P-value
Complications	Bleeding	1	0	2.143	0.829
	Hematoma	1	1		
	Infection	2	4		
	Seroma	2	3		
	Recurrences	1	1		

## Discussion

In this study, we compared NPWT to routine DDs after surgical excision of PSD. The main outcome of this research was the duration of wound healing after surgery. In terms of wound healing time, there was a statistically significant difference between the two groups, with the NPWT group needing less time. This also shortened the time for the NPWT group to resume everyday activities. The exclusion criterion of a 3 cm distance from the anal opening was established in this study. This decision was made based on the increased vulnerability of wounds located near the anal opening to infection, which can lead to a prolonged healing process. Moreover, pilonidal sinuses near the anal opening can present as perianal fistulas requiring fistulectomies as well. Along with this, patients with simple PSD or those who underwent flap surgery were excluded from the study. Studies on the effects of NPWT on wound management have been performed. Ubbink DT et al.'s systematic review, which included 13 randomized controlled trials investigating the use of NPWT in chronic and acute wounds as well as split skin grafts, found that NPWT did not result in faster wound healing when compared to control patients [13]. It concluded that there is little evidence to support the use of NPWT for wounds. However, a meta-analysis by Suissa D et al., which included 10 randomized controlled trials, found that NPWT resulted in quicker healing of chronic wounds than control [14]. The previously mentioned Cochrane Review concluded that the number of trials and patient numbers are lacking, and no rigorous, randomized, controlled trial offered evidence of the clinical effectiveness of NPWT [15]. However, when NPWT (77 patients) and DD modifications utilizing "moist wound dressings with alginates, hydrocolloids, foams, or hydrogels" (85 patients) were contrasted, faster healing was shown in a multicenter, randomized clinical trial [16]. The rate of granulation tissue formation was faster in the NPWT group than in the DD group (P=0.002), and wound closure was attained faster with NPWT than with DD (P=0.005). Forty-three (56%) NPWT patients finished healing compared to 33 (39%) in the DD group (P=0.04).

**Figure 4:**
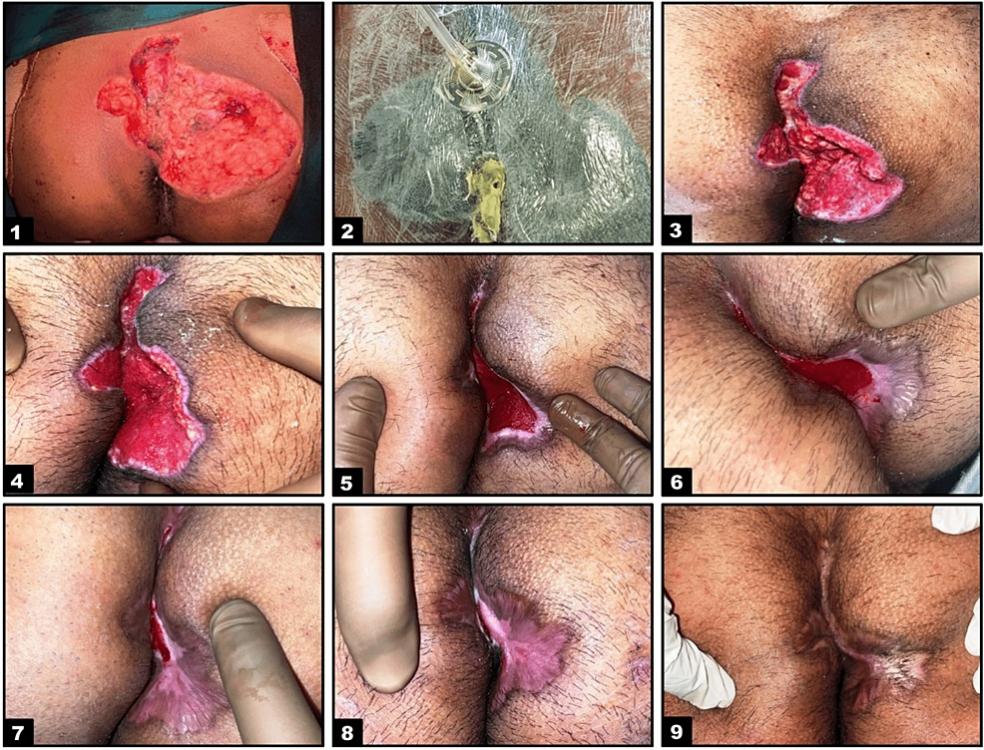
Wound healing via negative pressure wound therapy and epithelialization in a patient with pilonidal sinus disease following wide local excision.

Our study found that applying NPWT after surgery led to faster wound healing, with complete wound closure occurring between 40 and 81 days. Additionally, patients who received NPWT experienced significantly less pain 14 days after surgery. The most prevalent therapy for pilonidal disease is surgical excision and open wound healing. We think that NPWT would benefit patients in this group. The reason for the lack of use of NPWT is not a lack of knowledge or capability but rather a lack of access to NPWT tools and supplies, which is linked to a lack of reimbursement for this type of treatment [17]. Skin grafting, secondary suturing, and epithelialization are some of the alternatives available for wound healing after PSD wound bed preparation. The choice of approach relies on several variables, including the size and depth of the wound, the existence of an infection, and the availability of resources. Each of these procedures has benefits as well as drawbacks. Skin grafting works well to promote quick wound healing and lower the chance of recurrence. A quick and efficient procedure that may be used in an outpatient environment is secondary suturing. In addition to other modalities, DD is a straightforward and affordable approach to wound healing that involves migrating epithelial cells across the wound bed to form new tissue (Figure 4).

NPWT has been found to have a significant impact on wound maturation duration, allowing for secondary treatment and early wound closure. NPWT with instillation, a modified version of NPWT, has emerged as an effective approach for wound cleansing when used in conjunction with negative pressure. This innovative technique aids in managing infected wounds by facilitating the removal of infected tissue, thereby expediting the healing process [18]. While previous studies have investigated the use of NPWT with instillation utilizing saline solution, our study did not incorporate this method, highlighting the need for further investigation to assess its efficacy in treating infected PSD. In addition to the shorter wound preparation time with NPWT compared to DD techniques, our patients reported greater comfort using NPWT. This was attributed to the reduced pain they experienced during DD procedures, as observed during their follow-up visits.

The study was carried out in a single institution within a state, reducing patient caseload. Furthermore, there may be variations in the extent of pilonidal lesions among patients. Also, the individual response may vary with respect to wound dressings. To evaluate the enduring benefits of utilizing NPWT during the postoperative phase, it is recommended that future studies concentrate on patient preferences and quality of life throughout the different stages of wound healing, including long-term follow-up of healed patients.

## Conclusions

NPWT has been proven to be a better choice for wound management in the postoperative period of PSD treatment. It has quicker healing times, and patients can return to work faster than those treated with routine DDs. Hence, NPWT may be a viable alternative treatment option for complex PSD; however, additional research is necessary to assess its long-term outcomes and cost-effectiveness.
